# Associations of Maternal Prenatal Zinc Consumption with Infant Brain Tissue Organization and Neurodevelopmental Outcomes

**DOI:** 10.3390/nu17020303

**Published:** 2025-01-16

**Authors:** Paige K. Berger, Ravi Bansal, Siddhant Sawardekar, Catherine Monk, Bradley S. Peterson

**Affiliations:** 1 Department of Pediatrics, Brigham and Women’s Hospital, Boston, MA 02115, USA; 2 Department of Pediatrics, Harvard Medical School, Boston, MA 02115, USA; 3 Department of Psychiatry and Behavioral Sciences, Keck School of Medicine, University of Southern California, Los Angeles, CA 90033, USA; rabansal@chla.usc.edu; 4 Division of Child & Adolescent Psychiatry, The Saban Research Institute, Children’s Hospital Los Angeles, Los Angeles, CA 90027, USA; ssawardekar@chla.usc.edu; 5 Departments of Obstetrics and Gynecology and Psychiatry, Columbia University Medical Center, New York, NY 10032, USA; cem31@cumc.columbia.edu

**Keywords:** pregnancy, diet, nutrition, zinc, brain development, magnetic resonance imaging, infant

## Abstract

Background/Objectives: While studies in rat pups suggest that early zinc exposure is critical for optimal brain structure and function, associations of prenatal zinc intake with measures of brain development in infants are unknown. This study aimed to assess the associations of maternal zinc intake during pregnancy with MRI measures of brain tissue microstructure and neurodevelopmental outcomes, as well as to determine whether MRI measures of the brain mediated the relationship between maternal zinc intake and neurodevelopmental indices. Methods: Forty-one adolescent mothers were recruited for a longitudinal study during pregnancy. Maternal zinc intake was assessed during the third trimester of pregnancy using a 24 h dietary recall. Infant MRI scans were acquired at 3 weeks postpartum using a 3.0 Tesla scanner to measure fractional anisotropy (FA) and mean diffusivity (MD). Cognitive, language, and motor skills were assessed at 4, 14, and 24 months postpartum using the Bayley Scales of Infant Development. Results: Greater prenatal zinc intake was associated with reduced FA in cortical gray matter, particularly in the frontal lobe [medial superior frontal gyrus; β (95% CI) = −1.0 (−1.5, −0.5)], in developing white matter, and in subcortical gray matter nuclei. Greater prenatal zinc intake was associated with reduced MD in cortical gray matter and developing white matter [superior longitudinal fasciculus; −4.4 (−7.1, −1.7)]. Greater maternal zinc intake also was associated with higher cognitive development scores at 14 [0.1 (0.0, 0.1)] and 24 [0.1 (0.0, 0.2)] months of age; MRI indices of FA and MD did not mediate this relationship. Conclusions: Maternal prenatal zinc intake was associated with more favorable measures of brain tissue microstructural maturation and cognitive development during infancy.

## 1. Introduction

It has been more than 30 years since the Food and Nutrition Board of the National Academies reviewed the scientific evidence on maternal nutrition in pregnancy and lactation to establish guidelines for dietary intake that best supports infant growth and development [1]. In the intervening years, advances in technology, changes in the food environment, and new studies on individual nutrients have reaffirmed the importance of maternal nutrition to support optimal infant brain development [2,3,4,5,6]. Those studies provide new insights into dietary components that may be lacking, and they will inform updates to the national guidelines. For example, zinc has emerged as a nutrient of interest in the pre- and postnatal periods of human development for several reasons: (1) the current food environment favors calorie-dense foods that displace nutrient-dense alternatives containing zinc [7]; (2) the recommended dietary allowance (RDA) for zinc is highest in pregnancy and lactation because the physiological demand for zinc is highest then [8]; and (3) the physiological demand for zinc likely derives from an essential role it plays in the structural organization and metabolism of the infant brain [9].

Prior studies have shown that pre- and postnatal exposures to zinc are associated with later neurodevelopmental outcomes in infants and children [9,10,11]. For example, higher dietary zinc intake in the third trimester of pregnancy associates with better infant motor skills and habituation [11], and maternal zinc supplementation in late pregnancy associates with more fetal movement [12]. Animal studies have helped narrow the critical window for zinc exposure in humans, which translates approximately to the third trimester of pregnancy through the first month of lactation in infants. Rat dams fed a zinc-supplemented diet during late gestation through early lactation yielded rat pups with better spatial learning and memory compared to controls, and those fed a zinc-deficient diet produced pups with poorer cognitive outcomes [13]. The effects of early zinc exposure on neurodevelopmental outcomes may derive from effects of zinc on the development of the amygdala–hippocampus complex and cerebellum. In the amygdala and hippocampus, zinc is concentrated in synaptic vesicles, boutons, and mossy fibers, where it regulates storage and release of neurotransmitters [14]. In the cerebellum, zinc may enhance myelin compaction via its interaction with myelin basic protein [15,16,17,18,19,20].

No study has examined the influence of early zinc exposure on characteristics of brain development in newborn infants, which may drive the reported benefits of early zinc exposure on later neurodevelopmental outcomes. Therefore, the aim of this study was to assess associations of maternal zinc consumption in the third trimester of pregnancy with MRI indices of brain tissue microstructure in early infancy and neurodevelopmental outcomes during later infancy within the same cohort. The present study also explored whether the relationship between maternal zinc consumption and neurodevelopmental outcomes was mediated by MRI measures of the brain. The MRI modalities used in this study included diffusion tensor imaging (DTI), a technique that measures the diffusion of water molecules as influenced by brain tissue microstructure, and arterial spin labeling (ASL), which quantifies regional cerebral blood flow (rCBF), reflecting brain metabolism [4,6,21]. Neurodevelopment was measured using the Bayley Scales of Infant Development [22].

## 2. Materials and Methods

### 2.1. Participants

Participants were 41 adolescent mothers, and their infants recruited during pregnancy from the Departments of Obstetrics and Gynecology at Columbia University Medical Center (CUMC) and Weill Cornell Medical College. Mothers were eligible for inclusion if they attended routine prenatal appointments, had no significant health condition at the time of enrollment, had a 5th grade reading comprehension level, and were adolescents 14 to 19 years of age at the time of delivery [6]. Adolescents have the highest RDA for zinc compared to other age groups, particularly during pregnancy and lactation [23]. Mothers were excluded for reported use of drugs, tobacco, alcohol, or psychotropic medications and for any clinical diagnosis of fetal abnormalities. The Institutional Review Board at New York State Psychiatric Institute and Columbia University Medical Center approved study procedures. All participants provided written informed consent prior to data collection [3,6].

### 2.2. Study Design

Participant health information was abstracted from medical records and included maternal health related to their current pregnancy and infant characteristics at birth. Mothers completed three assessment visits throughout pregnancy (i.e., one visit per trimester), at which time information about dietary intake was collected using 24 h dietary recalls. Mothers returned to the laboratory with their infants at approximately 3 weeks of age, when infants underwent MRI scanning procedures, and again at 4, 14, and 24 months of age, when infants completed neurodevelopmental assessments [6].

### 2.3. Dietary Intake

Maternal dietary intake was assessed using the Automated Self-Administered 24-h (ASA-24) Dietary Assessment Tool, a web-based questionnaire that collects food and beverage intake from the prior 24 h [6]. The ASA-24 relies on scale images of portion sizes to assist the user in estimating servings consumed during each eating episode. ASA-24 then quantifies nutrients consumed from the previous day using three databases [6,24]. The primary exposure was dietary zinc intake, measured during each trimester of pregnancy. To account for other dietary factors that may influence newborn brain development, energy intake was included as a covariate in all statistical models. Energy intake serves as a proxy for overall dietary quality and nutrient adequacy. By adjusting for energy intake, this study aimed to isolate the specific effects of dietary zinc from those of a generally nutritious diet [25].

Although maternal dietary intake was assessed throughout pregnancy, this study reports herein maternal zinc intake during the third trimester for several reasons. Human studies have generally reported associations of dietary and supplemental zinc intakes during the third trimester with early neurodevelopmental outcomes, demonstrating their influences on enhancement of motor skill development in infants [11,12,26]. The third trimester is also characterized by rapid changes in brain tissue microstructure, including acceleration of circuit formation—these cellular events are the bases for neurodevelopmental outcomes and may be affected by zinc during the third trimester [27].

### 2.4. MRI Scanning Procedures

MRI scanning was completed within 3 weeks of birth. Newborn infants were fed, swaddled, and given time to fall asleep on the MRI scanning bed. Infants were not sedated, and all imaging data were collected without the use of contrast agent. Ear plugs and ear shields were applied to reduce noise from the MRI scanner. MRI-compatible EKG leads and a pulse oximetry sensor were affixed to the infant to monitor heart rate and oxygen saturation throughout MRI scanning. Recordings of MRI scanning sounds were used as ambient noise prior to the start of each pulse sequence to reduce waking and alerting in response to the sudden noise from the MRI scanner.

Table 1 provides a glossary of technical terms for the MRI scanning and processing procedures employed. Infant brain images were obtained using a 3.0 Tesla General Electric Signa MRI scanner. DTI data were used to compute fractional anisotropy (FA) and mean diffusivity (MD) for the direction and rate of the diffusion of free water molecules, which is modulated by brain tissue microstructure. FA values are indicative of directional water diffusion constrained by axons, and in areas of developing white matter, are an indication of the degree of myelination. MD measures average diffusion of water molecules, reflecting how extracellular matrix constrains the movement of water molecules. ASL was used to compute rCBF, a surrogate measure of brain metabolism, because blood flow and metabolism are tightly coupled.

DTI data were obtained in axial oblique slices parallel to the anterior–posterior commissure line using a single-shot echo planar DTI imaging sequence, with repetition time = 13,925 ms, echo time = 74 ms, field of view = 19 × 19 cm^2^, flip = 90°, acquisition matrix = 132 × 128 (acceleration factor = 2) zero-padded to 256 × 256 for 60 oblique axial contiguous slices positioned parallel to the anterior-posterior commissure line, and slice thickness = 2.0 mm. Three baseline images were acquired with b = 0 s/mm^2^ and 11 diffusion-weighted images at b = 600 s/mm^2^, with diffusion gradients applied in 11 directions sampling 3D space uniformly.

The ASL data were collected in axial oblique slices parallel to the anterior–posterior commissure line by employing a Pulsed Arterial Spin Labeling pulse sequence using QUIPSS II (Quantitative Imaging of Perfusion using a Single Subtraction) and PICORE (Proximal Inversion with Control for Off-Resonance Effects) [33]. A 9 cm tagging slab was placed 16 mm below the proximal edge of the imaging volume. The control images were acquired by applying off-resonance adiabatic hyperbolic secant RF pulse with the same frequency offset as that for the labeled images without the slice-selective gradient. Time to QUIPSS saturation ${TI}_{1}$ = 500 ms and inversion time of the first slice ${TI}_{2}$ = 1100 ms were used within a single-shot gradient-echo echo planar imaging (EPI) sequence with the following parameters: TR = 2300 ms, TE = 26.5 ms; matrix = 64 × 64; FOV = 19 cm, slice thickness = 5 mm, slice spacing = 0.5 mm, number of volumes acquired = 151; flip angle = 90°, scan time = 5 min 47 s. Sixteen to 18 slices were collected to ensure whole brain coverage.

A separate M_0_ scan was also collected within the same slices as the ASL data using a gradient-echo echo planar imaging (EPI) pulse sequence with parameters TR = 15,000 ms, TE = 25 ms; matrix = 64 × 64; FOV = 19 cm, slice thickness = 5 mm, slice spacing = 0.5 mm, flip angle = 90°, scan time = 15 s. We offline calculated M_0wm_ within white matter from these maps, which was used for quantification of rCBF maps.

ASL Overlay: T2-weigthed MR images with high in-plane resolution were collected in the same axial slice locations as the ASL data for normalizing ASL data with T2-weighted anatomical MR images. A 2D, fast spin echo pulse sequence was employed to collect these images: TR = 3500 ms, TE = 100 ms; matrix = 256 × 160; FOV = 19 cm, slice thickness = 5 mm, slice spacing = 0.5 mm, echo train length = 24, flip angle = 90°, scan time = 1 min.

### 2.5. MRI Processing Procedures

DTI: Magnetic field inhomogeneities, eddy-current distortions, and head motion were corrected using an in-house programs toolbox. An ellipsoid was then fitted to the 11 gradient directions and 3 baseline diffusion-weighted imaging data using a Levenberg–Marquardt algorithm while constraining the diffusion tensor to be positive definite. From the diffusion tensors fitted at each voxel in the image, mean diffusivity (MD) and fractional anisotropy (FA) maps were computed for a diffusion tensor D with eigenvalues $\left(\lambda_{1},\lambda_{2},\lambda_{3}\right)$ ordered such that $\lambda_{1}>\lambda_{2}\geq \lambda_{3}$ [30], $FA=\frac{\sqrt{{\left(\lambda_{1}-\hat{\lambda }\right)}^{2}+{\left(\lambda_{2}-\hat{\lambda }\right)}^{2}+{\left(\lambda_{3}-\hat{\lambda }\right)}^{2}}}{\sqrt{2\left(\lambda_{1}^{2}+\lambda_{2}^{2}+\lambda_{3}^{2}\right)}}$, and $MD=\hat{\lambda }=\frac{\lambda_{1}+\lambda_{2}+\lambda_{3}}{3}$. The maps for FA and MD values were spatially normalized using a rigid body similarity transformation to the template brain. A nonlinear spatial warping of FA and MD maps into the template brain was then computed using a method based on fluid dynamics [34].

The template brain was selected using a 2-step procedure that ensured findings were not unduly influenced by the specific brain selected as the template. First, a preliminary template was selected as the brain of one infant whose postmenstrual age and overall brain size were nearest to the group averages. Postmenstrual age at the time of MRI scan is defined as the time that elapsed between the mother’s last menstrual period and birth of the infant, also termed gestational age at birth, plus chronological age from birth to the time of MRI scan [32]. The brains for all other infants were spatially coregistered to that preliminary template, and the distance of each point on the surface of each brain from the corresponding point on the preliminary template brain was measured. The brain for which all points across its surface were closest (in the least squares sense) to the average distance was selected as the final template. The final template brain therefore was morphologically most representative of all infant brains.

ASL: within-participant head motion was corrected by aligning all PASL images and the M_0_WM_ image to the first PASL image. The aligned images were spatially smoothed using a Gaussian kernel of 6 mm FWHM (full width at half maximum), which improved signal-to-noise ratio. The mean PASL image was computed and used as a mask to remove nonbrain tissue. Subsequently, a voxel-wise map of rCBF from the PASL time series and an M_0_WM_ image using in-house software were developed: (1) the control images were pairwise subtracted from the labeled images, (2) and then the average of the subtracted images was computed. The rCBF value at each voxel was quantified as $rCBF=\frac{6000*∆I}{2\alpha *M_{0\_B}*{TI}_{1}*exp\left(-{TI}_{2}/T_{1\_B}\right)}$, where ∆I is the image difference obtained in step (2); α = 0.9 is the tagging efficiency; T_1B_ = 1664 ms is the T_1_ of blood [35]; M_0_B_ is the MR signal from a voxel filled with arterial blood, estimated from the M_0_WM_ map as $M_{0\_B}=rM_{0\_WM}e^{(1/T_{2WM}-1/T_{2B})TE}$, where *r* = 1.06 is the proton density ratio of blood; and T_2WM_ = 70 ms and T_2B_ = 200 ms [33,36,37].

Each participant ASL map was rigidly coregistered (3 rotations and 3 translations) into its T2-weighted MR image by maximizing the mutual information between the map and image [38]. The anatomical MR image was similarity transformed (3 rotations, 3 translations, and 1 global scale) to the template brain. This similar transform was applied to the coregistered ASL map for normalizing ASL maps for all participants into the coordinate space of the same template brain. All statistical analyses were conducted at each voxel of the brain in template space.

### 2.6. Neurodevelopmental Assessment

The Bayley Scales of Infant Development (Third Edition) were used to assess cognitive, language, and motor skill scores at 4, 14, and 24 months [22]. The cognitive scale measures sensorimotor integration, concept formation, attention, habituation, and memory. The language scale assesses both receptive and expressive language skills. The motor scale evaluates fine and gross motor skills. Scaled scores were used as the primary outcome variables for all domains. Scaled scores are based on raw scores transformed to reflect the average performance of a normative sample at a given age, corresponding to a set position on a normal distribution curve. The internal consistency of the cognitive, language, and motor skill scale ranges is from 0.86 to 0.91 [22].

### 2.7. Statistical Analysis

Descriptive statistics are reported as mean ± standard deviation or as percentages. Multiple linear regression applied to each voxel of the image was used to determine the significance of the correlation coefficient for maternal prenatal intake of zinc (mg/day) with infant FA, MD, and rCBF values at 3 weeks of age. Maternal intake of energy (kcal/day), postmenstrual age (PMA) at the time of the MRI scan, infant sex, and birthweight were included as covariates in all analyses. False positives in the testing of multiple hypotheses were controlled by assessing the spatial extent of clusters of significant findings and computing the probability of one or more clusters occurring by chance, using a cluster-defining Student’s t-statistic of 2.5. *p*-values for clusters that survived the multiple hypothesis testing procedure were color-coded and displayed on the template brain. All statistical maps were constructed using in-house software. Regions of interest were identified with an age-specific DTI atlas for the infant brain.

Whether infant MRI values (M) mediated the association of maternal prenatal zinc intake (X) with infant neurodevelopmental outcomes (Y) was assessed at each brain voxel using three regression models: (1) Y = i_1_ + cX + e_1_, estimating the total effect (c) of maternal zinc intake on neurodevelopmental outcomes; (2) Y = i_2_ + c′X + bM + e_2_, estimating the direct effect (c’) of maternal zinc intake on neurodevelopmental outcomes while accounting for infant MRI values (M); and (3) M = i_3_ + aX + e_3_, estimating the association (a) of maternal zinc intake with infant MRI values. Here, i_1_, i_2_, and i_3_ are intercepts; c, c′, a, and b are coefficients; and e_1_, e_2_, and e_3_ are error terms. Covariates in each of the 3 regressions were PMA, infant sex, and birthweight. We then tested whether the indirect effect a×b differed significantly from zero using a z-score, $z_{ab}=(a\times b)/{se}_{ab}$, where ${se}_{ab}=\sqrt{\left(a^{2}\times {se}_{b}^{2}\right)+(b^{2}\times {se}_{a}^{2})}$, and ${se}_{a}$, ${se}_{b}$ were the standard errors of the regression coefficients a and b, respectively [39,40]. *p*-values for the statistical significance of the mediation effects at each brain voxel were then displayed on the template brain.

## 3. Results

**Figure 1:**
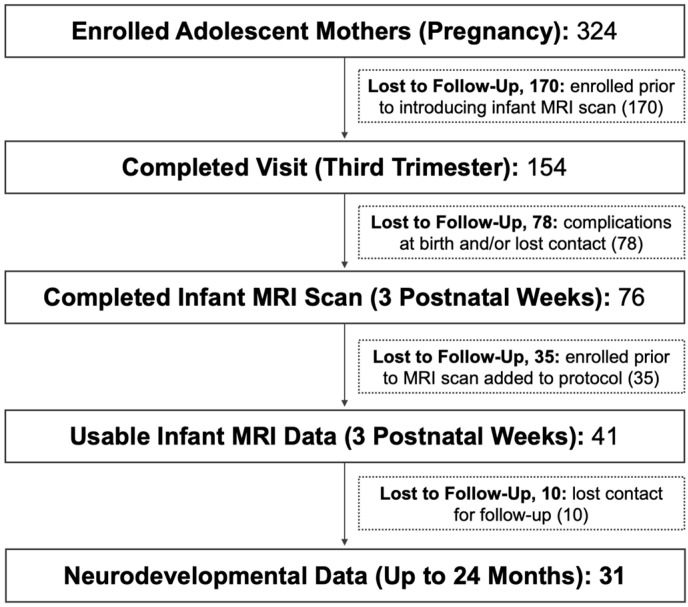
Participant flow chart.

Characteristics of mother–infant pairs are presented in Table 2. Forty-one infants, who were enrolled after MRI scanning procedures were added to the parent study and who had usable MRI data collected within 3 weeks of age were included in analyses related to brain tissue microstructure and regional metabolism (Figure 1). Of those infants, 31 returned for neurodevelopmental testing during later infancy. All mothers self-identified as Latina. Infants were born full-term (i.e., ≥37 weeks of gestation) and with normal birthweight (i.e., ≥2500 g). Proportions of males and females were similar. Mothers reported consuming, on average, 25.1 ± 19.3 mg of zinc/day in the first trimester, 29.0 ± 18.0 mg/day in the second trimester, and 24.4 ± 15.0 mg/day in the third trimester. Additionally, 35%, 32%, and 32% of participants reported prenatal zinc intakes below the RDA for zinc during the first, second, and third trimesters, respectively, defined as 12 mg/day for females ages 14–18 years old who are not pregnant [23].

**Figure 2:**
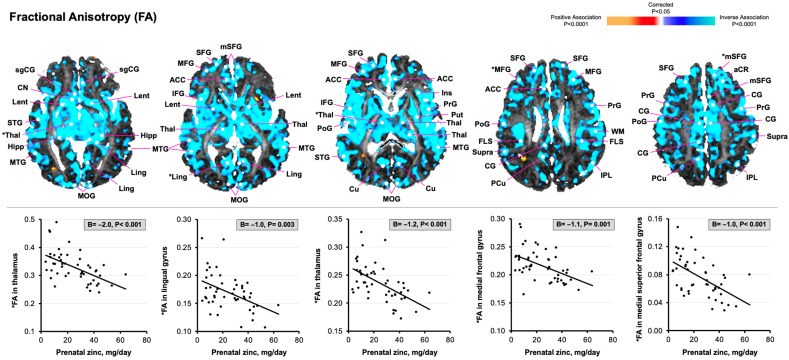
Statistical maps for the associations of maternal zinc consumption during the third trimester of pregnancy with newborn FA values. The statistical significance of the associations between maternal zinc intake and diffusion-derived FA values are color-coded, and only *p*-values that survived FDR correction are plotted. The voxels in blue indicate reduced FA values in widespread cortical gray matter regions and subcortical gray matter nuclei. Specifically, the scatterplots highlight significant inverse associations of maternal zinc intake with FA values in the medial superior frontal gyrus, medial frontal gyrus, thalamus, and lingual gyrus [denoted by asterisk (*) in brain maps]. Abbreviations: aCR, anterior corona radiata; ACC, anterior cingulate cortex; CN, caudate nucleus; Cu, cuneus; FA, fractional anisotropy; FLS, superior longitudinal fasciculus; FOF, occipito-frontal fasciculus; Hipp, hippocampus; IC, internal capsule; IFG, inferior frontal gyrus; IPL, inferior parietal lobule; Ins, insular cortex; Lent, lenticular nucleus; Ling, lingual gyrus; MFG, medial frontal gyrus; MOG, medial occipital gyrus; MTG, medial temporal gyrus; mSFG, medial superior frontal gyrus; O_WM_, occipital white matter; PCu, precuneus; PoG, postcentral gyrus; PrG, precentral gyrus; Put, putamen; superior frontal gyrus; sgCG, subgenual cingulate gyrus; STG, superior temporal gyrus; Supra, supramarginal gyrus; Thal, thalamus.

Maternal zinc intake during each trimester of pregnancy was consistently associated with newborn brain tissue microstructure and rCBF in the same regions and tissue types. Herein, this study reports the associations between maternal zinc intake during the third trimester and MRI outcomes within 3 weeks of age. As shown in Figure 2, greater maternal zinc intake was associated with reduced FA values in widespread cortical gray matter regions, predominantly in the frontal lobe [e.g., medial superior frontal gyrus, β (95% CI) = −1.0 (−1.5, −0.5); medial frontal gyrus, −1.1 (−1.7, −0.5)] and in temporal cortices [e.g., medial temporal gyrus, −1.0 (−1.5, −0.6)]. Maternal zinc intake also associated inversely with FA values in posterior parietal and occipital regions that included the medial occipital gyrus [−0.9 (−1.4, −0.4)] and in subcortical gray matter nuclei, such as the thalamus [−1.2 (−1.8, −0.6)] and basal ganglia [−1.4 (−2.2, −0.7)].

**Figure 3:**
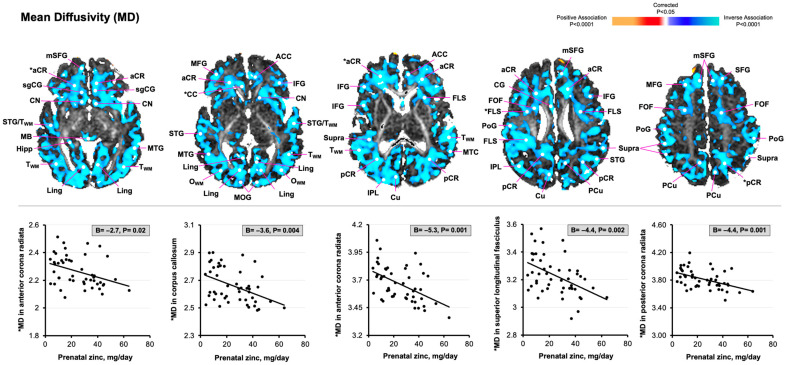
Statistical maps for the associations of maternal zinc consumption during the third trimester of pregnancy with newborn MD values. As similarly described in Figure 2, statistically significant associations between maternal zinc intake and diffusion-derived MD values are color-coded, and only *p*-values that survived FDR correction are plotted. The voxels in blue denote reduced MD values in areas of developing white matter throughout the brain. The scatterplots depict significant inverse associations of maternal zinc intake with MD values in the posterior corona radiata, superior longitudinal fasciculus, anterior corona radiata, and corpus callosum [denoted by asterisk (*) in brain maps]. Abbreviations: aCR, anterior corona radiata; ACC, anterior cingulate cortex; CN, caudate nucleus; Cu, cuneus; FLS, superior longitudinal fasciculus; FOF, occipito-frontal fasciculus; Hipp, hippocampus; IFG, inferior frontal gyrus; IPL, inferior parietal lobule; Lent, lenticular nucleus; Ling, lingual gyrus; MB, midbrain; MD, mean diffusivity; MFG, medial frontal gyrus; MOG, medial occipital gyrus; MTG, medial temporal gyrus; mSFG, medial superior frontal gyrus; O_WM_, occipital white matter; pCR, posterior corona radiata; PCu, precuneus; PoG, postcentral gyrus; Put, putamen; SFG, superior frontal gyrus; sgCG, subgenual cingulate gyrus; STG, superior temporal gyrus; Supra, supramarginal gyrus; T_WM_, temporal white matter; WM, white matter.

**Figure 4:**
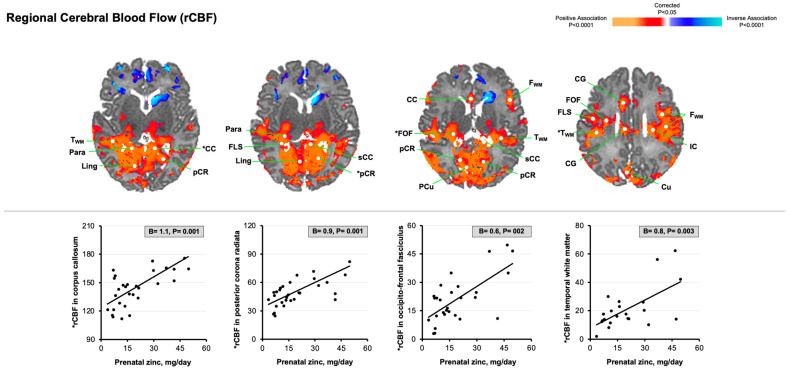
Statistical maps for the associations of maternal zinc consumption during the third trimester of pregnancy with newborn rCBF values. The statistical significance of the associations of maternal zinc intake with ASL-derived rCBF values at each point on the surface of the brain is color-coded, with voxels in red denoting increased rCBF values and voxels in blue denoting decreased rCBF values. Only *p*-values that survived FDR correction are plotted. The scatterplots highlight significant positive associations of maternal zinc intake with rCBF values in temporal white matter, occipito-frontal fasciculus, posterior corona radiata, and corpus callosum [denoted by asterisk (*) in brain maps]. Abbreviations: CG, cingulate gyrus; Cu, cuneus; FLS, superior longitudinal fasciculus; FOF, occipito-frontal fasciculus; F_WM_, frontal white matter; IC, internal capsule; Ling, lingual gyrus; Para, parahippocampus; pCR, posterior corona radiata; PCu, precuneus; sCC, splenium of the corpus callosum; STG, superior temporal gyrus; T_WM_, temporal white matter.

Greater maternal zinc intake was associated with reduced MD values in cortical gray matter and developing white matter throughout the brain, including in the fronto-occipital fasciculus [−3.9 (−7.6, −0.3)], superior longitudinal fasciculus [−4.4 (−7.1, −1.7)], posterior corona radiata [−4.4 (−7.0, −1.8)], and corpus callosum [−3.6 (−5.9, −1.2)] (Figure 3). As shown in Figure 4, greater maternal zinc intake was associated with increased rCBF values in the middle and posterior regions of developing white matter, in similar locations where there were reduced MD values [e.g., fronto-occipital fasciculus, 0.6 (0.3, 1.0); posterior corona radiata, 0.9 (0.4, 1.4); corpus callosum, 1.1 (0.5, 1.7)].

Maternal zinc intake during the third trimester was not significantly associated with cognitive development scores at 4 months of age [0.05 (−0.1, 0.2)]; however, positive associations of maternal zinc intake with cognitive development scores emerged at 14 [0.12 (0.0, 0.2)] and 24 [0.11 (0.0, 0.2)] months of age. MRI-derived FA and MD values did not mediate the association of maternal zinc intake with cognitive outcomes. Maternal zinc intake did not associate significantly with scores for language or motor skill development at 4, 14, or 24 months of age.

## 4. Discussion

Maternal consumption of zinc during pregnancy was associated significantly and diffusely with MRI indices of newborn brain tissue microstructure and regional metabolism. Specifically, maternal zinc intake during the third trimester was associated with reduced FA values in the frontal, temporal, and cingulate cortices and in subcortical gray matter nuclei. The reported intake of zinc also was associated with reduced MD values within regions of developing white matter, including the fronto-occipital fasciculus, superior longitudinal fasciculus, and posterior corona radiata, and with increased rCBF values within these same regions of developing white matter. Additionally, maternal zinc intake during the third trimester was associated with better cognitive development in later infancy, when more complex cognitive capacities emerge. Collectively, the data suggest that early zinc exposure has multifaceted effects on newborn brain maturation in diverse regions and tissue types, with potential sustained benefits for cognitive function up to 24 months.

Although many environmental factors influence early brain maturation, prenatal nutrition is a modifiable target for optimizing future neurodevelopmental outcomes. Prenatal zinc intake is an especially promising candidate because of its dual role in supporting maternal and fetal health [41,42,43,44]. Prenatal zinc intake mitigates inflammation and oxidative stress associated with birth complications, and it fosters a favorable intrauterine environment for fetal brain maturation that supports future cognitive capacities [41,43,44]. Many pregnant women may not meet recommendations for dietary zinc intake [45], and higher zinc intake may benefit neurodevelopmental outcomes in their babies. For example, a longitudinal study in Egyptian women found that 54% consumed <9 mg/day of dietary zinc during pregnancy, and the infants of those who had greater dietary zinc intake had better habituation scores at 6 months of age [11]. A clinical trial in Peruvian women reported that supplementation with zinc (15 mg/day) increased serum zinc concentrations during pregnancy (and thus indicated at least mild deficiency), and it increased fetal activity levels at 36 weeks’ gestation [12]. Similarly, the present study found that one-third of participants had less than the recommended prenatal zinc intake, and yet prenatal zinc intake was associated positively with cognitive development up to 24 months of age. These findings underscore the importance of prenatal zinc nutrition for infant outcomes.

While the observed associations of prenatal zinc intake with infant neurodevelopmental outcomes align with those in previous reports, the present study is the first to report associations of maternal zinc intake during pregnancy with MRI indices of newborn brain maturation, specifically in regions involved in (but not limited to) cognitive and motor control (e.g., frontal, cingulate, and motor cortices; thalamus; and basal ganglia). Maternal zinc intake was associated inversely with FA values, predominantly in the frontal cortex and in subcortical gray matter nuclei. Previous neuroimaging studies in infants have shown that FA values decline in the cortex after 32 weeks’ gestation, and animal studies suggest that this age-related decline in FA values reflects characteristics of cortical gray matter (e.g., dendritic arborization, synapse formation) [46,47]. One seminal study in fetal baboons found that FA values in the cortex declined with advancing gestational age, reflecting the maturational processes of dendritic arborization and synaptogenesis [46,47]. Given that FA is an index of water diffusion with directional preference, and that dendrites and the synapses they support arborize in all directions without directional specificity, findings suggest that maternal zinc intake in pregnancy may promote dendritic arborization and synapse formation in cortical and subcortical gray matter nuclei [48].

The present study also found that the reported maternal intake of zinc was associated inversely with MD values in regions of cortical gray matter and developing white matter throughout the newborn brain. Prior studies in infants have shown that MD values decline with age in areas of developing white matter, indicating that restrictions on the diffusion of water perpendicular to the direction of axons within white matter increase with age [49,50,51]. When the age-related decline in MD values are accompanied by an increase in FA values in the same locations (representing greater diffusion of water parallel to the direction of axons), those age effects likely represent progressive myelination [51,52]. However, no increase in FA values in developing white matter was observed in association with increasing maternal zinc intake during the third trimester. Therefore, the association of third trimester maternal zinc intake with lower MD values in regions of developing white matter likely represents an effect of zinc on developmental processes other than myelination, such as increasing axon size or density [51,52].

Additionally, maternal intake of zinc was positively associated with rCBF values in developing white matter, in similar locations to where significant associations with MD values were observed. rCBF is tightly coupled with regional brain metabolism, including the glucose utilization and oxygen consumption that support the energy demands of the developing brain [53]. rCBF values may be higher in regions of developing white matter where MD values are lower because increasing axonal density increases metabolic demands and a more rapid delivery of oxygen and glucose [54].

Collectively, the results of this study suggest that maternal intake of zinc during pregnancy influences newborn brain tissue microstructure and regional metabolism through several mechanisms, including greater dendritic arborization and synapse formation, as well as increased axonal density. Consistent with these interpretations, zinc is distributed in synaptic vesicles of glutamatergic neurons, which are concentrated in the cortex, amygdala, and hippocampus, especially in lamina containing dendrites and synapses [55,56,57]. Zinc has many biological and physiological roles in those neurons, which affect brain excitability and synaptic plasticity. For example, zinc regulates receptors on postsynaptic neurons, inhibiting N-methyl-D-aspartate and GABA receptors [58,59,60,61,62], and it activates signal transduction pathways inside postsynaptic neurons [63], including protein kinase c, calcium/calmodulin-dependent kinase II, and tropomyosin-related kinase receptors [58,64]. These zinc-stimulated activities may increase long-term potentiation in the frontal cortex and hippocampus, strengthening synaptic connections to support future learning and memory [65,66]. Given these physiological functions, it follows that inverse associations of maternal zinc intake with FA values were observed in the frontal cortex and subcortical gray matter nuclei, including the amygdala–hippocampus complex, lending support to the hypothesized role of zinc in synaptic plasticity in these regions [14,63].

Another mechanism through which maternal zinc intake during pregnancy may produce the observed associations with diffusion indices in the newborn brain is through the accumulation of neurofilament proteins and regulation of motor proteins, which move along microtubules of axons to influence axonal transport. For example, zinc stimulates signal transduction pathways, including the mitogen-activated protein kinase cascade, which phosphorylates neurofilament proteins [58,67,68,69,70]. Phosphorylated neurofilament proteins are the most abundant and space-filling structures within microtubules of axons, and they slow axonal transport due to prolonged pauses in neurofilament movement [71,72,73]. Moreover, zinc blocks the microtubule binding of microtubule-associated proteins, inhibiting the activity of motor proteins that transport cargoes along axons, including neurofilament proteins [74,75]. Zinc-induced phosphorylation of neurofilament proteins and inhibition of motor proteins, which contribute to slowed axonal transport, may explain in part the observed associations of maternal zinc intake with reduced MD values within areas of long fiber bundles. Neurofilament accumulation and axonal transport are important for calibrating conduction speed and coordinating signals across long axons, which supports faster information processing in the brain [72].

This study had several limitations. The prospective observational design does not support causal inferences. Randomized controlled trials are needed to manipulate maternal diet and zinc intake to discern the causal effects of zinc on newborn brain development. Such studies should expand their focus from adolescent Latina mothers (a population with rates of zinc deficiency as high as 56%) to racially and ethnically diverse cohorts in geographically distinct locations [76,77,78]. Assessment of maternal zinc consumption in this study was based on a 24 h dietary recall rather than direct observation, and the study did not collect biological specimens to assess more reliable measures of maternal zinc nutriture or status. Contrary to our hypothesis, MRI indices of infant brain maturation did not significantly mediate the association of maternal zinc intake with cognitive outcomes. Nevertheless, maternal zinc intake did associate with both MRI indices and cognitive outcomes, and MRI measures associated significantly with cognitive outcomes, suggesting that failure to detect significant mediation effects was likely attributable to a modest sample size and limited statistical power [79]. Larger studies are needed to test mediation effects and confirm the observed associations of zinc intake with neurodevelopmental indices.

Overall, the findings of this study highlight the essential role of maternal zinc intake during pregnancy on diffusion-derived indices of the newborn infant brain and support the use of neuroimaging to provide novel insights into the effects of maternal nutrition on brain tissue microstructure and regional metabolism. In addition, the findings suggest sustained benefits of in utero exposure to zinc on cognitive function during later infancy. The findings of this study may be especially relevant to vulnerable infants, such as those born prematurely [44]. Although this study found that maternal zinc intake throughout pregnancy is important for early brain development, 60% of fetal zinc is acquired during the third trimester, after many preterm infants are already born, increasing their risk for zinc deficiency [80,81]. Although studies have shown that zinc supplementation in preterm infants may reduce morbidity and mortality and increase growth [26,80,82], neuroimaging studies are needed to determine the influences of zinc supplementation on structural maturation of the brain.

## 5. Conclusions

Overall, zinc is an essential nutrient in the developing infant brain. Early exposure may play a critical role in neurobiological processes, such as dendritic arborization, synaptogenesis, and axonal growth, which are the structural bases for future neurodevelopmental outcomes. Randomized controlled trials are needed to establish the causal effects of maternal zinc supplementation on newborn brain maturation, potentially guiding more precise prenatal nutrition guidelines.

## Figures and Tables

**Table 1:** Glossary of magnetic resonance imaging (MRI) technical terms and methods.

Technical Term	Abbreviation	Definition
Arterial spin labeling	ASL	Magnetic resonance imaging modality that measures regional cerebral blood flow [28].
Diffusion tensor imaging	DTI	Magnetic resonance imaging modality that measures the diffusion of water in the brain as influenced by characteristics of brain tissue microstructure. Those characteristics are indexed by fractional anisotropy and mean diffusivity [27,29].
Fractional anisotropy	FA	Diffusion tensor-imaging-derived index of the degree to which the diffusion of water molecules is preferentially constrained in a particular direction [29].
Full width at half maximum	FWHM	Image processing measure that indicates the extent of smoothing applied to an image [30].
Levenberg–Marquardt algorithm		Mathematical optimization method used in fitting diffusion tensor models to diffusion tensor imaging data [31].
Magnetic resonance imaging	MRI	Non-invasive imaging technology that produces three-dimensional anatomical images of soft tissues in the body, including the brain.
Mean diffusivity	MD	Diffusion tensor-imaging-derived index of the overall rate diffusion of water molecules averaged across all three spatial directions [29].
Myelination		Process by which axons within fiber bundles are wrapped in myelin, a fatty sheath that increases the speed of neural transmission [27].
Postmenstrual age	PMA	Time elapsed between the first day of the last menstrual cycle and birth (gestational age) plus the time elapsed after birth (chronological age) [32].
Regional cerebral blood flow	rCBF	Arterial-spin-labeling-derived measure of blood flow in a voxel [28].
Template brain		Reference brain used to spatially transform, co-register, and standardize brain images across different participants.
Voxel		Three-dimensional unit in an imaging dataset that represents a small volume of tissue within the scanned area; it contains information about tissue properties at a specific location.

**Table 2:** Characteristics of mother–infant dyads.

	Prenatal Zinc Cohort (N = 41)	
	Mean	SD
Mothers		
Age at delivery (years)	18.2	1.4
Pre-pregnancy BMI (kg/m^2^)	25.2	6.4
Hispanic ethnicity (%)	95.0	
Kilocalories per day, average (kcals)	2459	1049
Zinc per day, average (mg)	24.4	15
Infants		
Female (%)	39	–
Birth weight (g)	3204	456
Postmenstrual age at MRI scan (days)	42.6	1.7

Values are mean ± SD or %.

## Data Availability

Data described in the manuscript, code book, and analytic code will be made available upon request pending application and approval from the authors due to ethical reasons.
